# Unraveling the mechanisms of propofol-induced psychological dependence: a multi-omics approach linked to gut microbiota in hippocampal function

**DOI:** 10.3389/fmed.2025.1539467

**Published:** 2025-04-03

**Authors:** Li Wang, Tangyi Wang, Yadian Lei, Yudong Su, Yuxin Lin, Zhijing Wu, Qiong Wu, Shoude Zhang, Haiyan Wang

**Affiliations:** ^1^Department of Basic Medical Sciences, Qinghai University Medical College, Xining, Qinghai, China; ^2^State Key Laboratory of Plateau Ecology and Agriculture, Qinghai University, Xining, Qinghai, China; ^3^Research Center for High Altitude Medicine, Qinghai University, Xining, Qinghai, China; ^4^Key Laboratory of the Ministry of High Altitude Medicine, Qinghai University, Xining, Qinghai, China

**Keywords:** propofol, psychiatric dependence, gut microbes, transcriptomics, metabolomics, hippocampus

## Abstract

**Introduction:**

Drug abuse is becoming a global public health crisis. According to the United Nations, the number of drug users worldwide has increased dramatically over the past decade, with a surge in the number of drug abusers. The problem was exacerbated by the expanding market for illicit drugs and the increasing availability of synthetic drugs such as fentanyl. Clinical drug abuse is a problem that requires particular attention, and the potential addictive properties of some drugs and their mechanisms of action are currently unknown, which limits the development and implementation of drug addiction intervention strategies.

**Methods:**

Eight-week-old C57BL/6J mice were used as study subjects. A mental dependence model was established using the conditional position preference experiment (CPP), and the hippocampal tissues of the model mice were subjected to RNA-seq transcriptome sequencing, LC–MS non-targeted metabolome sequencing, and intestinal macro-genome sequencing in order to discover propofol mental dependence signature genes. Correlation analyses of transcriptomics and metabolomics were performed using the Spearman method, and gene-metabolite networks were mapped using Cytoscape software. Real-time fluorescence quantitative PCR and immunoprotein blotting (Western blotting) methods were used to validate the characterized genes.

**Results:**

After the conditioned position preference experiment, the conditioned preference scores of the 75 mg/kg propofol and 2 g/kg alcohol groups were significantly higher than those of the control saline group. 152 differential genes and 214 differential metabolites were identified in the 75 mg/kg group. Cluster analysis revealed that changes in the neuroactive ligand receptor pathway were most pronounced. Gut microbiomics assays revealed significant changes in five differential enterobacterial phyla (*Campylobacter phylum*, *Thick-walled phylum*, *Anaplasma phylum*, *Actinobacteria phylum*, and *Chlorella verticillata phylum*) in the 75 mg/kg propofol group, which may be related to changes in the differential expression of dopamine.

**Discussion:**

These findings suggest that 75 mg/kg propofol has a significant mind-dependent effect on the biology of drug addiction through neuroactive ligand-receptor interaction pathways in conjunction with the tricarboxylic acid cycle, and the metabolic pathways of alanine, aspartate, and glutamate that may influence intestinal microbial changes through bidirectional signaling.

## 1 Introduction

Propofol, as a fast-acting short-acting anesthetic, is widely used in a variety of clinical practices, including short-term anesthesia for abortion, gastroscopy, and the induction and maintenance of general anesthesia (1), but also for conscious sedation in critically ill patients, as well as in the treatment of refractory agitated delirium and antiemetic. Recent studies have also shown that propofol has therapeutic and anti-inflammatory effects (2). Its main mechanism of action is to inhibit neural signaling by promoting chloride inward flow via GABA-A type receptors (3). However, the risk of propofol abuse and addiction cannot be ignored, especially among medical professionals (4, 5), long-term or overdose use of propofol may lead to dependence, increased tolerance, and withdrawal symptoms, and overdose may even be life-threatening (6).

Drug addiction is considered a chronic and relapsing brain disorder characterized by persistent craving for and use of drugs regardless of negative consequences (7). Underlying this craving and use behavior are long-term gene expression changes, neuronal adaptations, and changes in synaptic plasticity triggered by repeated drug ingestion. The hippocampus, a key brain region for learning and memory, plays a central role in drug addiction (8). Drug addiction can lead to significant changes in neuroplasticity in the hippocampus that include changes in neuronal excitability, neurotransmission, morphological changes in dendrites and axons, and synapse formation or elimination (9). Neurotransmitter systems in the hippocampus, including dopamine, glutamate, and GABA, are closely linked to the neural mechanisms of drug addiction (10). Current research on the mechanisms of drug addiction has focused on changes in brain neurotransmitters (dopamine, glutamate, and GABA, among others) (11). The hippocampal region, a key brain area for learning, memory, and spatial navigation, plays a central role in the development and maintenance of drug addiction (8). Addictive drugs such as cocaine, opioids, and nicotine alter the structure and gene expression of the region by modulating synaptic plasticity in the hippocampus. In addition, drug abuse-induced changes in neurotransmitter systems, including serotonin and endorphins, have been strongly associated with the development of addictive behaviors by modulating mood, memory, and reward behaviors. Most addictive drugs are directly linked to reward effects by increasing the release of dopamine in the brain, particularly in the hippocampus, leading to craving and dependence on the drug (12). In neurobiological models of addiction, changes in dopamine receptor expression in the hippocampus have received widespread attention, e.g., chronic drug exposure (e.g., cocaine, endogenous cannabinoid analogs) may lead to down-regulation of dopamine D2 receptors in the hippocampus, which may be related to reduced sensitivity to drugs and disinterest in non-pharmacological rewards in addicted individuals (13). Dysregulated dopamine signaling in the hippocampus has also been associated with the risk of relapse in drug addiction, as these alterations may affect an individual’s response to drug-related cues and decision-making processes. Thus, the hippocampus and dopamine system play a critical role in the development, maintenance, and relapse of drug addiction, and long-term alterations in these brain regions provide a neurobiological basis for the persistence of addiction and the complexity of treatment.

Multi-omics technology refers to the integrated application of various genomics techniques such as genomics, transcriptomics, proteomics, metabolomics, etc., to comprehensively analyze the changes in biological samples at different biological levels, and in the case of drug addiction, many researchers have used metabolomics to find that metabolites such as (inositol-1-phosphate, free fatty acids, and metabolites related to tricarboxylic acid cycle, etc.) are increased in the brain of rats after heroin addiction (14). Microbiome and metabolomics approaches to study methamphetamine users identify microbial metabolic pathways involved in addiction (15). The effects of chronic methamphetamine exposure on the neural proteome in the hippocampus and olfactory bulb region of rats were also investigated by proteomic approaches, revealing significant changes in the expression of 18 proteins related to addiction such as (synaptic vesicle glycoprotein 2A, myelin proteolipoproteins, etc.) (16). These technologies enable us to probe deeply into the biological basis of drug addiction at the molecular level, revealing the underlying gene expression regulation, protein function changes, metabolic pathway remodeling, and complex networks of inter-cellular interactions. For example, the development of single-cell sequencing technology and spatial transcriptomics provides a powerful tool to study the cellular heterogeneity and tissue microenvironment of drug addiction (17, 18). Dysregulation of gut flora in alcohol addiction and modification of addiction using gut flora modification (19). The application of these technologies not only greatly broadens our understanding of the pathogenesis of drug addiction, but also provides a solid scientific basis for the development of new treatment strategies. At present, the combined application of propofol and multi-omics technology is still in its infancy, and the related research results are limited. Transcriptome studies have found that propofol can change the structure and function of the developing heart, suggesting its potential cardiotoxic effect (20). Metabolomics studies have shown that propofol can significantly increase the level of inflammatory marker glycoprotein acetylation (GlycA) (21).

In recent years, the interaction between gut microbiota and host metabolism has become a hot topic in biomedical research, such as the brain-gut axis and the gut-liver axis. The gut microbiome not only plays a key role in maintaining the host’s nutritional metabolism, immune regulation and intestinal barrier function, but also affects the host’s systemic metabolic status through its metabolites (22). More and more evidence suggests that the interaction between gut microbiome and host metabolism may play an important role in the pathogenesis of drug addiction. On the one hand, gut microbes can affect the behavior and mood of the host by regulating neurotransmitter levels and immune responses. On the other hand, metabolic changes may reflect the systemic effects of drug abuse on the body.

**Figure 1:**
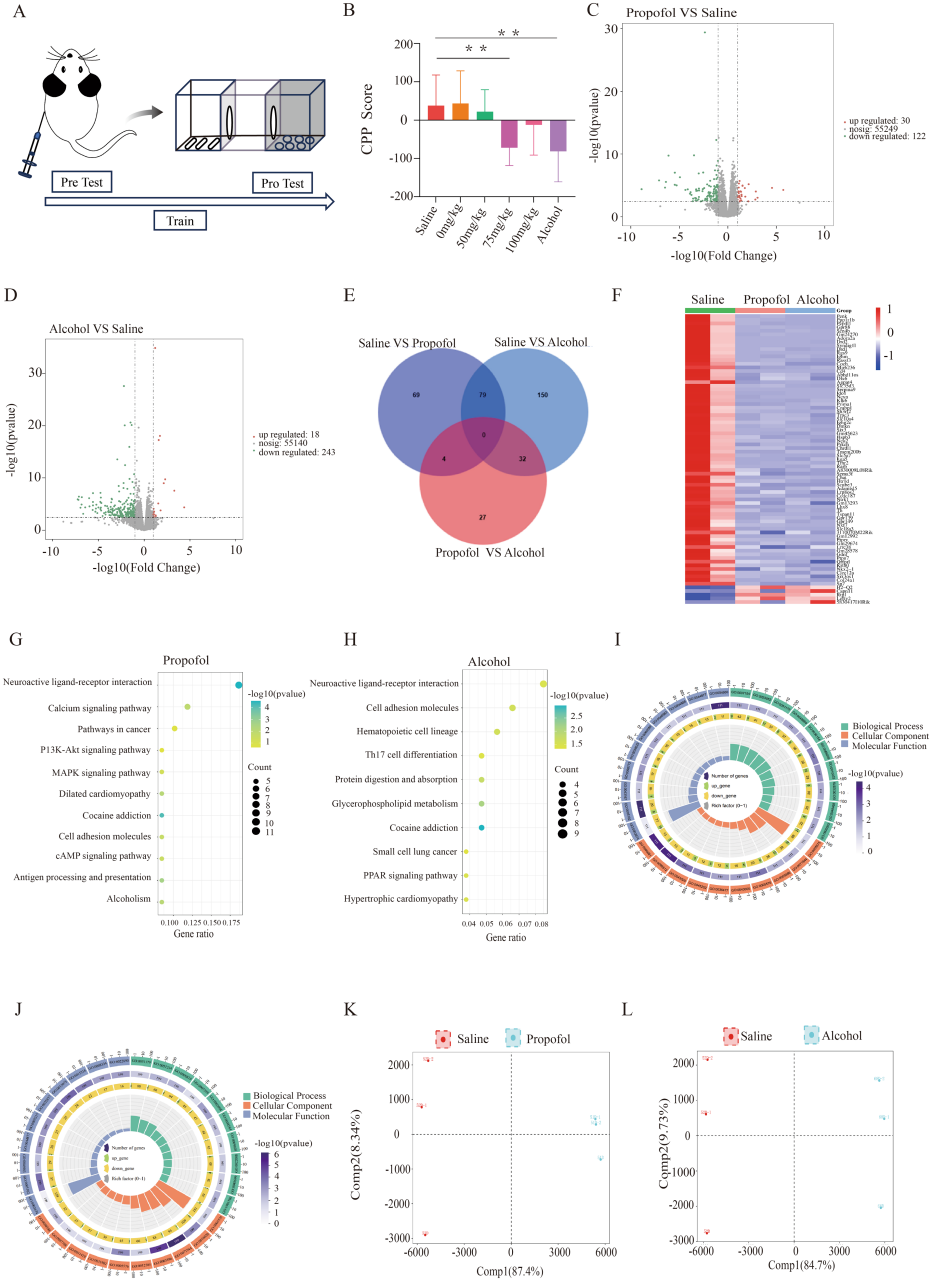
Hippocampal transcriptome changes in 75 mg/kg propofol-addicted mice. **(A)** Study design. **(B)** Change in preference scores for each group after the conditional location preference experiment (preference preference score = time before the experiment - time after the experiment) *p* < 0.05, *p < 0.01. **(C)** Volcano map of the propofol group. **(D)** Volcanic map of the alcohol group. **(E)** Differential gene intersections between the propofol group and the saline and alcohol groups. **(F)** Heat map of gene expression in saline, propofol group and alcohol group. **(G)** KEGG-enriched bubble map of the propofol group. **(H)** KEGG-enriched bubble plot for the alcohol group. **(I)** GO analysis enrichment circle plot for the propofol group. **(J)** GO analysis enrichment circle plot for the alcohol group. **(K)** Metabolite principal component analysis plot for the propofol group. **(L)** Metabolite principal component analysis plot for the alcohol group.

The purpose of this study is to explore the mechanism of propophenol-induced mental dependence in mice by network pharmacology, transcriptomtics, metabolomics and metagenomics, reveal the molecular mechanism of propofol addiction, explore the changes of hippocampal and intestinal flora in mice after propofol addiction, and provide therapeutic strategies for clinical treatment of propofol addiction and prevention of propofol abuse. The flow chart of the experiment is shown in Figure 1A.

## 2 Materials and methods

### 2.1 Chemical reagents

Propofol was purchased from McLean Biotechnology (D806979), HiScript II Q RT SuperMix for qPCR (R223-01, vazyme, Nanjing, China), primers were purchased from Xianghong Bio-technology Co., Ltd., and antibodies to DRD1 and DRD2 were purchased from Proteintech (17934-1-AP, Wuhan, China).

### 2.2 Animal models and experimental design

Healthy male C57BL/6J mice (6 weeks old, weighing 20–23 g) were purchased from Xi’an Fraser Biotechnology Co. The mice were acclimatized to the laboratory environment for 2 weeks before the experiment. Standard diet and water were ad libitum. 72 mice were randomly divided into 6 groups of 12 mice each, including saline control group, propofol-treated group (0 mg/kg, 50 mg/kg, 75 mg/kg, 100 mg/kg) and alcohol control group. Propofol was injected intraperitoneally and 100 mg/kg was chosen as the highest dose based on the literature that the 114 mg/kg dose resulted in the loss of the flip reflex in mice. The normal saline group and alcohol group were used as negative and positive controls, respectively. The experiment was divided into adaptation stage, training stage and testing stage. The adaptation period lasted for 3 days. Every day, the mice were placed in the middle channel, opened the channel door, and explored freely for 30 min to help the mice adapt to the experimental environment. On the fourth day, the basic time test was performed with a 15 min limit time, and the mouse’s residence time in the preference box and the non-preference box was recorded as the baseline data of behavioral preferences. Then enter the 20 day training phase. In the morning, mice were intraperitoneally injected with normal saline, propofol and alcohol of different concentrations, and placed in the preference box to close the door of the middle channel; In the afternoon, all mice received the same volume of normal saline injection as the control group, placed in the non preference box, closed the channel door, and then cycled to the 21st day, entered the test phase, reopened the door of the middle channel, the mice explored freely for 15 min, and recorded the residence time in the preference box and the non preference box. The preference score was calculated by the residence time before and after the experiment to evaluate the effect of propofol and alcohol on the behavioral preference of mice (23, 24). At the end of the test, mice were executed and hippocampal tissues were separated on ice and stored in liquid nitrogen for subsequent experiments. All experiments involving propofol drug addiction in this study were approved by the Ethics Committee of Qinghai University School of Medicine (2022–01). Animal experiments were conducted in accordance with the European Guidelines for the Care and Use of Laboratory Animals (2010/63/EU).

### 2.3 Metabolomics

A 50–100 mg sample was taken and added to a methanol-acetonitrile mixture for low temperature sonication extraction. Centrifuge at 12000 rpm for 10 min, take the supernatant and add 200 μL of 30% acetonitrile solution to re-dissolve and centrifuge at 14000 rpm for 15 min. Samples were analyzed using Vanquish UPLC (Thermo, USA). Samples were separated on a Waters HSS T3 column with electrospray ionization source detection. Raw data were pre-processed using Progenesis QI software (Waters Corporation, Milford, USA), normalized and imported into R software. The metabolic abundance of each group of samples was standardized to eliminate the technical variation between different samples. On this basis, the average abundance of each group of metabolites was calculated, and the FC value was further calculated. The *p* value was calculated by Student’s t-test, and the VIp value was calculated by multivariate statistical analysis method OPLS-DA. When screening differential metabolites, strict thresholds were set: P value 1, and FC > 1.5 or < 0.667. The metabolites that met these conditions were identified as differential metabolites, and the significance level of metabolite enrichment in each pathway was analyzed by Fisher’s exact test.

### 2.4 Transcriptomics

Total RNA was extracted by Trizol reagent and evaluated for quantity and purity. High-quality RNA samples were selected for construction of sequencing libraries. The mRNA was enriched using the magnetic bead method, followed by fragmentation, reverse transcription and PCR amplification. DESeq2 software was used to identify differentially expressed transcripts and genes, setting |log_2_(fold change)| >1 as the threshold, and KEGG and GO enrichment analyses were performed. *p* value corrected *p* < 0.05 was used as the screening criterion to further analyze the GO function and KEGG pathway enrichment.

### 2.5 Macrogenomics

Samples of intestinal contents were collected and total DNA was extracted from the samples and tested for DNA purity and integrity. DNA samples were broken and libraries were constructed for high-throughput sequencing. Raw data were pre-processed in Illumina fastq format to remove host contamination. The obtained sequences were spliced and assembled, and gene prediction, annotation and classification were performed. Finally, the samples were subjected to similarity clustering, sequencing tests and statistical comparison of differences. For the analysis of intestinal microbiota, principal component analysis (PCA) was used to preliminarily distinguish the significant differences between different groups. Subsequently, the differences in the composition of intestinal microbial communities under different groups were shown by species composition histograms, and these differences were further quantified by linear discriminant analysis (LDA). In order to identify microbial species with significant differences, STAMP analysis tool and Wilcoxon rank sum test were used to analyze the differences between groups, and the significance level was also set as *p* < 0.05. This method can not only identify different species, but also visually display their distribution in different groups.

### 2.6 Network pharmacology and molecular docking

Potential targets of propofol were collected from four databases, PharmMapper, SwissTargetPrediction, Drugbank and BATMAN-TCM, and from NCBI, GeneCard, Therapeutic Targets Database, Pharmacogenomics Knowledge Base four databases to collect relevant targets for drug addiction (25–28). The Wayne diagrams of propofol targets and drug addiction targets were drawn using the Microbiotics Online Platform.^1^ Protein interaction networks were constructed using the STRING database. The DAVID database was used to perform GO and KEGG pathway enrichment analyses and visualization of potential targets. The 3D structural data of propofol were downloaded from the PubChem database, while the protein structural data were obtained from the PDB database. Molecular docking was performed through the CB-Dock2 platform.^2^

### 2.7 Comprehensive analysis of transcriptomics and metabolomics

On the basis of transcriptomics and metabolomics sequencing analysis, the correlation analysis of OPLS-DA was performed on the differential genes and differential metabolites of propofol at a concentration of 75 mg / kg, and the load map was drawn. Subsequently, the differential metabolites and differential genes were analyzed by Pearson correlation analysis for clustering heat map drawing, and the correlation was set to *p* < 0.05 (*p* < 0.05, ‘*’) to explore the correlation between genes and metabolites. After that, Cytoscape (Cytoscape v3.9.0) was used to draw a gene-enzyme reaction-metabolite network diagram to explore the relationship between genes and metabolites.

### 2.8 Real-time fluorescence quantitative PCR

RNA was extracted from hippocampus, converted to cDNA by reverse transcription kit, and amplified by PCR using SYBR method. Through the PCR instrument, the fluorescence signal changes were monitored and collected to obtain the Ct value (cycling threshold), and the relative quantitative analysis was performed by the 2^^–ΔΔCt^ method.

### 2.9 Western blotting

Hippocampal tissue was mixed with RIPA lysate, protease and phosphatase inhibitor (100:1:1) and ground, and the supernatant was subjected to polypropylene gel electrophoresis, incubated overnight at 4°C with primary antibody against DRD1 and DRD2, and then the secondary antibody was incubated and developed, and the images were collected.

### 2.10 Statistical analysis

Behavioral data were calculated by the preference score formula, plotted and analyzed using GraphPad Prism software v10.1.2. Comparisons between multiple groups were analyzed using one-way ANOVA with *p* < 0.05 as the criterion for significance. All experiments were repeated three times with *p* < 0.05 as the criterion for statistical significance.

## 3 Results

### 3.1 Behavioral analysis

The results of CPP showed that the preference scores of mice in the 75 mg/kg Propofol group (Propofol group) changed significantly and at p < 0.05 compared to the saline control group, the preference scores of mice in the Alcohol group changed significantly and at *p* < 0.05 compared to the saline group, and the preference scores of mice in the Propofol group showed the same trend of change compared to the Alcohol group which indicated that isoPropofol did form a mental dependence, see (Figure 1B).

### 3.2 Transcriptomic analysis of propofol group and alcohol group

In the transcriptomic analyses performed in the propofol and alcohol groups, we used |log_2_FC| > 1 and p < 0.05 as the criteria for screening differentially expressed genes. The results showed that there were 152 genes with significant changes in expression in the propofol group, of which 30 were up-regulated and 122 were down-regulated. The alcohol group, on the other hand, had 261 genes with significant changes in expression, including 18 up-regulated and 243 down-regulated genes (Figures 1C,D). Cross-tabulation analysis of gene expression revealed 79 common differentially expressed genes. Clustering heatmap analysis further revealed that the propofol and alcohol groups were similar in differential gene expression patterns (Figures 1E,F). KEGG pathway enrichment analysis pointed out that samples from both groups exhibited significant changes in cocaine addiction and neuroactive ligand-receptor interaction pathways (Figures 1G,H). In addition, GO functional enrichment analysis identified 10 significantly enriched biological processes covering signal transduction, dopamine receptor activity, and lipid and organic acid binding functions (Figures 1I,J).

### 3.3 Metabolomic changes in propofol and alcohol groups

**Figure 2:**
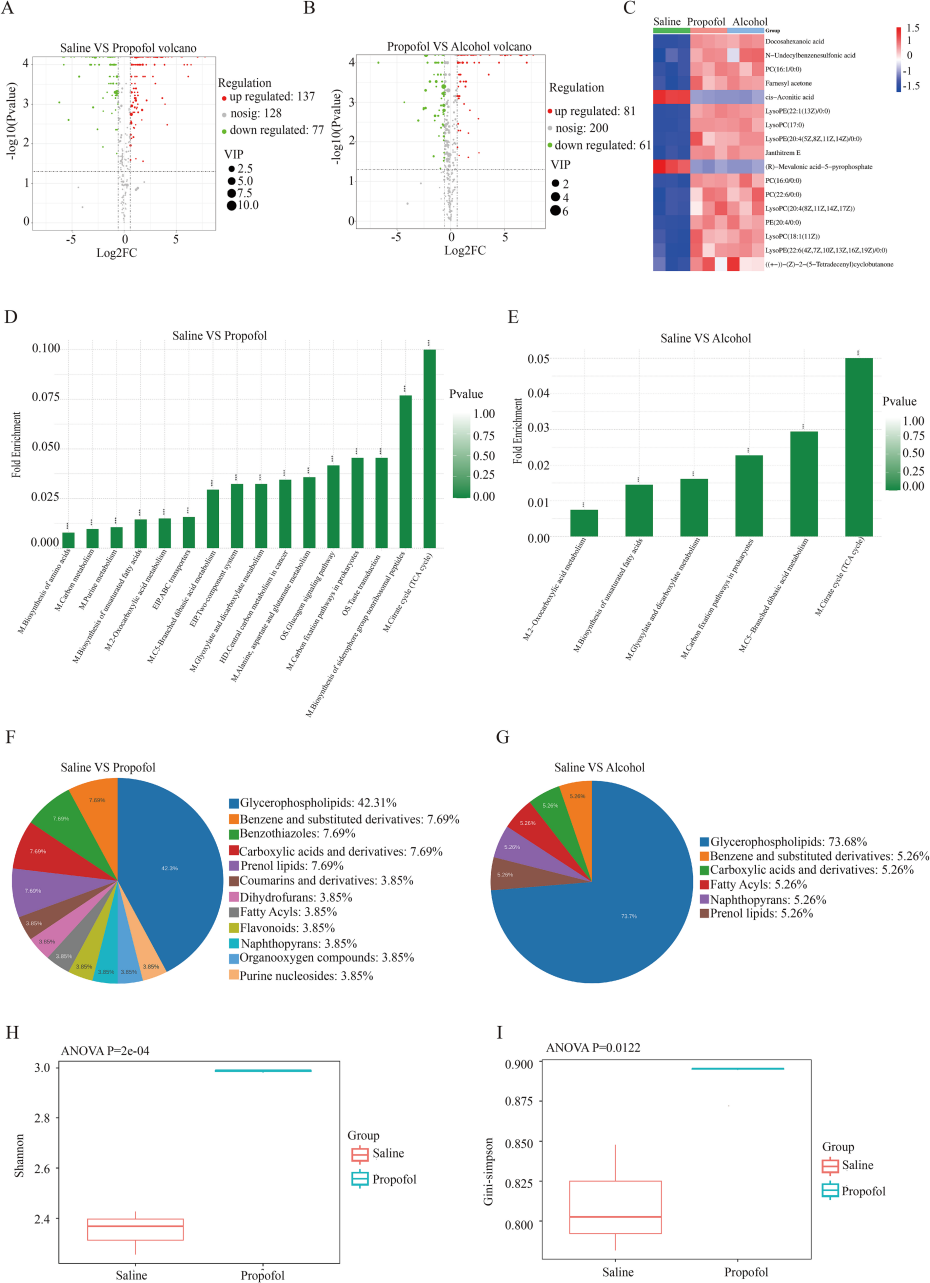
Changes in metabolomics in the hippocampus in the 75 mg/kg group. **(A)** Metabolite volcano plot of the propofol group. **(B)** Metabolite volcano plot of the propofol group versus the alcohol group. **(C)** Gene expression heatmap of the saline, propofol and alcohol groups. **(D)** KEGG pathway enrichment histogram for the propofol group. **(E)** KEGG pathway enrichment histogram for the alcohol group. **(F)** Pie chart of chemical classification of differential metabolites in the propofol group. **(G)** Pie chart of chemical classification of differential metabolites in the alcohol group. **(H)** Shannon index box plot of alpha diversity of gut microbes in the propofol group. **(I)** Box plot of the Gini-simpson index of alpha diversity of gut microbes in the propofol group.

Alterations in hippocampal metabolic profiles by propofol addiction were assessed from metabolite expression levels, and OPLS-DA analysis of the propofol and alcohol groups showed that samples within the propofol and alcohol groups clustered together, whereas the samples between the groups tended to be significantly separated, suggesting that there were significant differences between the groups (Figures 1K,L). Volcano plots of the differential metabolites screened in the propofol group and alcohol group are shown (Figures 2A,B). The cross-differential metabolite heatmaps of the saline, propofol, and alcohol groups clearly showed the differences in metabolites and 17 cross-differential metabolites between the propofol and alcohol groups and the control saline group (Figure 2C), which indicated that the metabolite trends were similar in the propofol and alcohol groups. Next, metabolite-related metabolic pathways were analyzed using the KEGG pathway library and plotted as bar graphs (Figures 2D,E), which showed that the metabolites in the propofol group and the alcohol group were mainly concentrated in the citric acid cycle, 2-oxocarboxylic acid metabolism, and alanine, aspartate, and glutamate metabolic pathways. In addition to the common metabolic compounds such as glycerophospholipids, carboxylic acids and their derivatives, which were found in the propofol and alcohol groups, there were also antioxidant and neuroprotective compounds such as benzothiazoles, coumarins and their derivatives, organic oxides, and purine nucleosides, as shown in the graphs (Figures 2F,G).

### 3.4 Differences in gut microbes between propofol and saline groups

**Figure 3:**
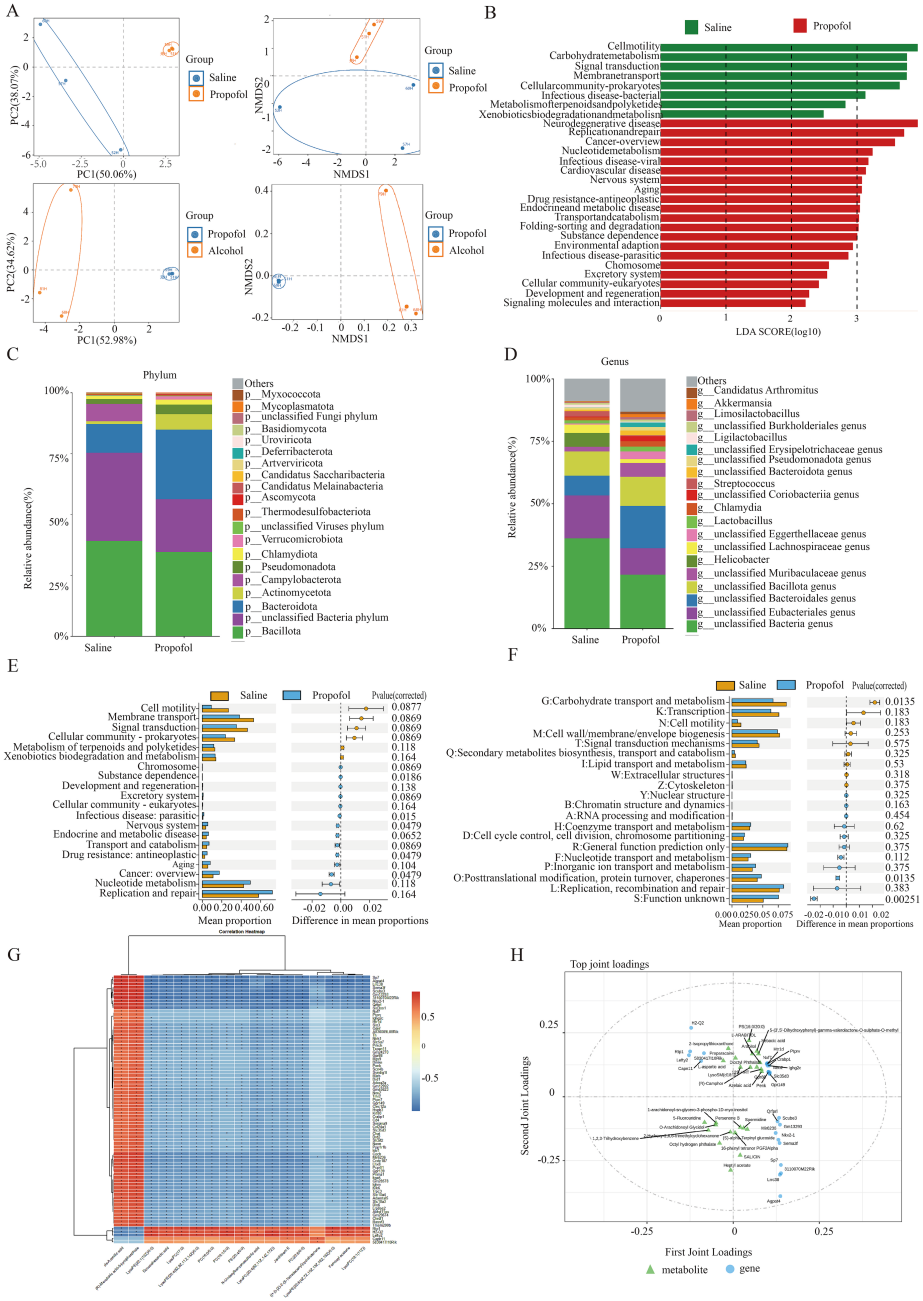
Characteristic gut microbiological changes in the propofol group and the control group. **(A)** PCA and NMDS analysis of gut microbes in the propofol group versus the saline and alcohol groups. **(B)** Linear discriminant analysis of gut microbial metabolic pathways in the propofol group. **(C)** Changes in species composition at the phylum level in the propofol group. **(D)** Species composition changes at the genus level in the propofol group. **(E)** KEGG pathway maps for significant differences in microbial abundance in the propofol group. **(F)** Functional annotation maps of eggNOG for significant differences in microbial abundance in the propofol group. **(G)** Correlation matrix heatmaps of differential genes with differential metabolites in the propofol group. *p < 0.05, **p < 0.01. **(H)** OPLS-DA analyses of differential genes with differential metabolites in the Top20.

To assess the effect of propofol addiction on gut microbial diversity, we analyzed species evenness and richness in the propofol group versus the saline group using Simpson’s index and Shannon’s index. The results showed that species evenness and richness were significantly higher in the propofol group than in the saline group (Figures 2H,I). Principal component analysis (PCA) and non-metric multidimensional scaling (NMDS) further revealed a high degree of clustering of the samples in the propofol group, indicative of a high diversity of community composition (Figure 3A). Linear discriminant analysis of LEfSe software identified potential biometabolic pathways (Figure 3B). Histogram analysis of species abundance revealed significant changes in microbial composition at the phylum level in the propofol group, including the disappearance of the Campylobacter phylum, a decrease in the thick-walled phylum, and an increase in the Anaplasma phylum, Actinobacteria phylum, Pseudomonas phylum, and Micrococcus wartyi phylum. At the genus level, *H. pylori* disappeared, Streptococcus decreased, and Lactobacillus spp. and Akkobacter spp. increased in abundance, changes indicative of key biomarker flora in the propofol group (Figures 3C,D). Comparative analysis of the macrogenomic data using Stamp software gave us information on the species composition abundance, functional prediction and their differences in the propofol group. KEGG and eggNOG functional prediction analyses revealed a significant increase in substance-dependent, neural and drug-resistance-associated metabolic pathways in the propofol group (Figures 3E,F).

### 3.5 Integrated analysis of metabolomics and transcriptomics data

**Figure 4:**
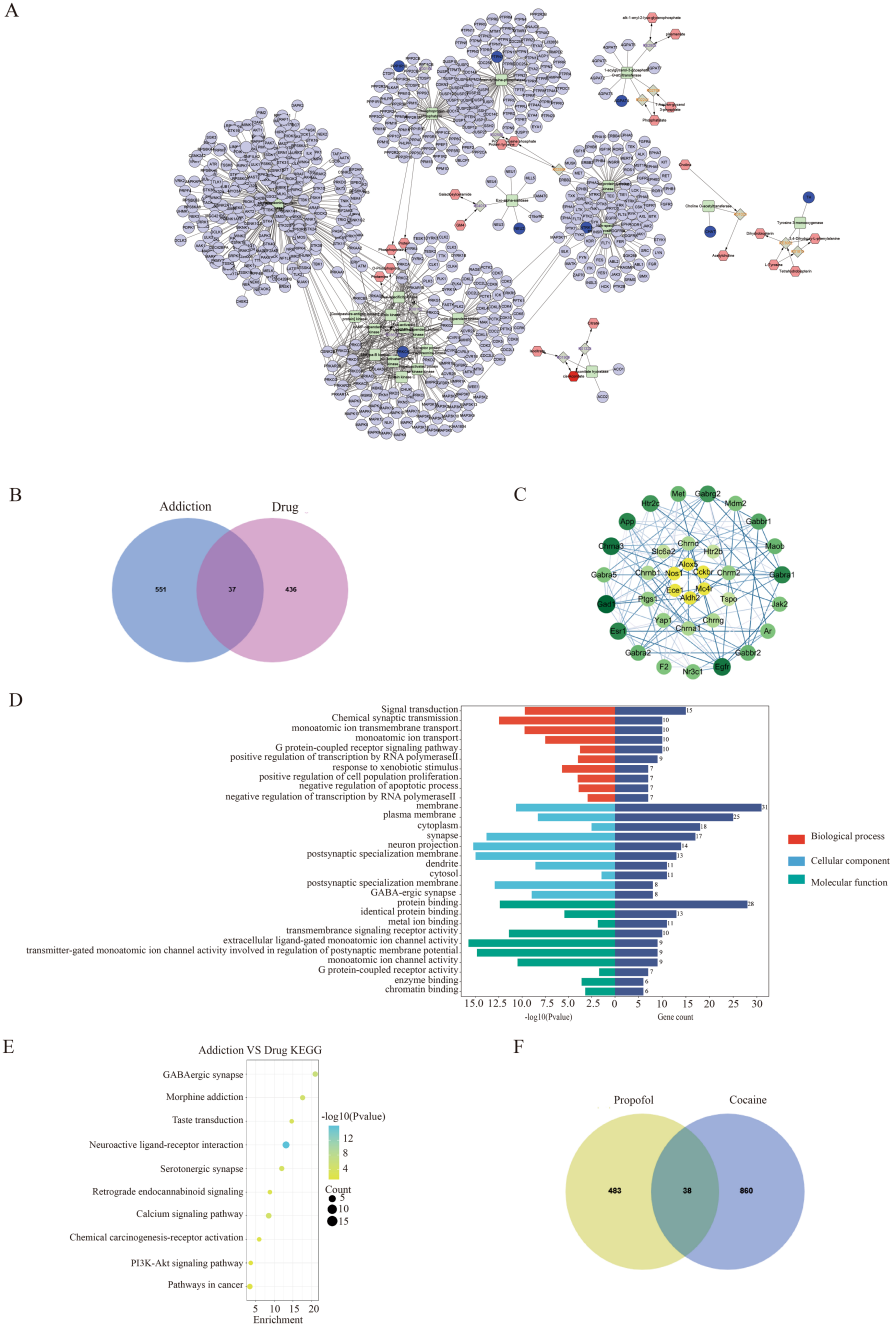
Network pharmacology and differential gene-metabolite association analysis of propofol and addiction. **(A)** Gene-metabolite network mapping with cytoscape. **(B)** Venn of targets of action of propofol versus targets of action of addiction. **(C)** Protein interactions map between propofol targets of action and targets of addiction. **(D)** GO enrichment analysis of the common targets of action of propofol and addiction. **(E)** KEGG pathway-enriched bubble map of the intersection of propofol targets of action and targets of addictive action. **(F)** Venn of propofol addiction targets versus cocaine addiction targets.

To gain a deeper understanding of the biological changes in the hippocampal region of the propofol group, we constructed a correlation matrix heat map of 79 common differential genes and 17 common differential metabolites among the saline, propofol and alcohol groups by Spearman correlation analysis (Figure 3G), which revealed the expression patterns of key genes and metabolites. The analysis revealed significant correlations (*p* < 0.05) between 1,062 pairs of differential genes and metabolites, e.g., the dopamine receptor was positively correlated with cis-aconitine and (R)-ergosterol-5-pyrophosphate, and negatively correlated with docosahexaenoic acid (DHA), a metabolite of lysophosphatidylethanolamine, and lysophosphatidylcholine. In addition, OPLS-DA analysis of Top25 differential genes and metabolites further revealed the correlation between them (Figure 3H). The compound-reaction-enzyme-gene network diagram constructed using Cytoscape 3.9.0 software (Figure 4A) revealed the potential interactions and pathway regulation between metabolites and genes. These findings not only elucidated the biological changes in the propofol group, but also provided important data for the discovery of therapeutic targets and biomarkers for propofol addiction.

### 3.6 Network pharmacology and molecular docking analysis

**Figure 5:**
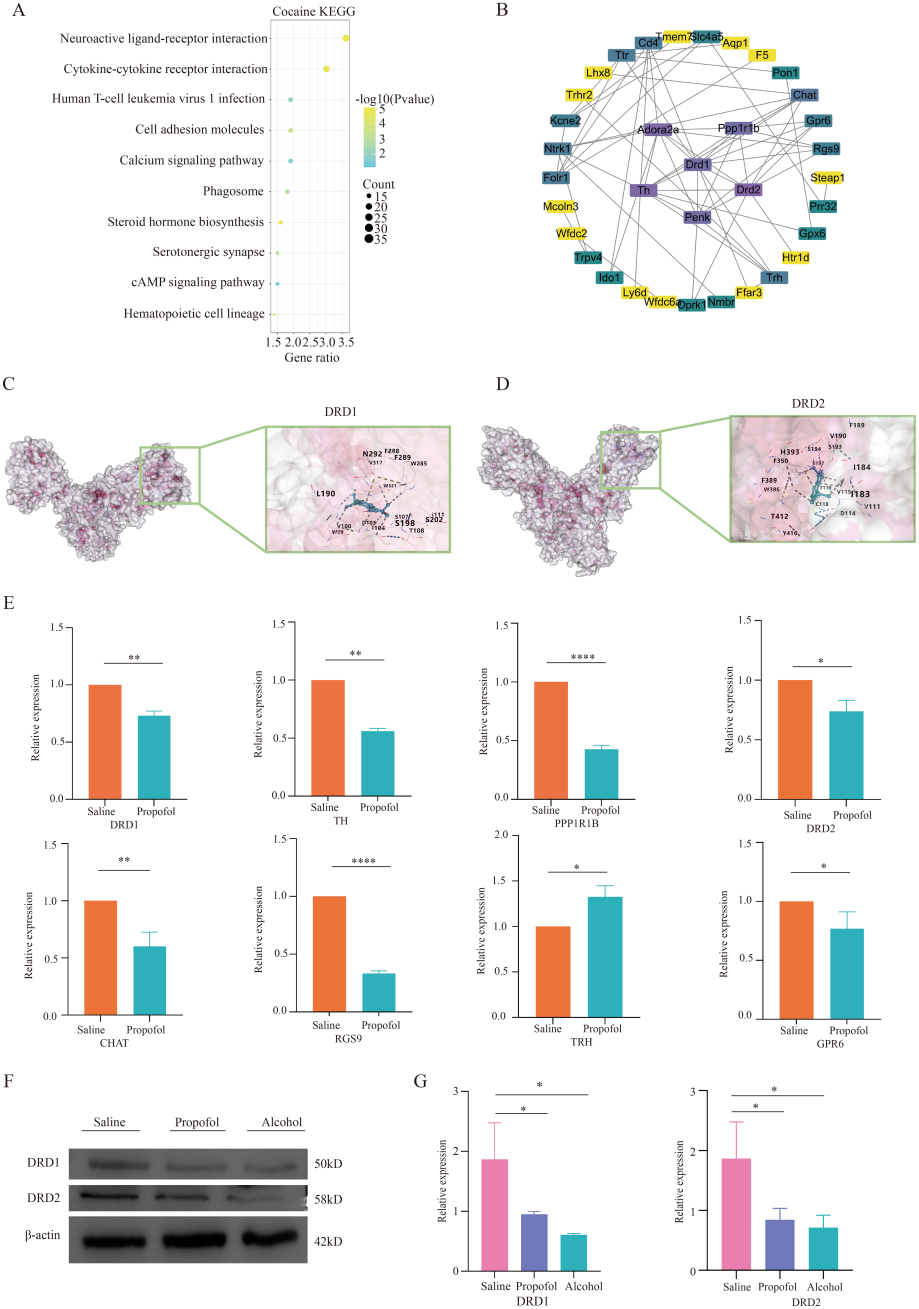
Validation of differential gene correlations in the neuroactive receptor-ligand interaction pathway. **(A)** KEGG-enriched bubble plot of cocaine addiction targets. **(B)** Protein interaction plots of genes on the neuroactive receptor-ligand pathway. **(C)** Diagram of molecular docking models for dopamine receptor 1. **(D)** Diagram of molecular docking models for dopamine receptor 2. **(E)** QPCR validation of differential genes in significant pathways regarding propofol addiction. *p < 0.05, *p <* 0.01 and *p <* 0.001 and ***p < 0.0001. **(F,G)** Western blotting validation of dopamine receptor 1 and dopamine receptor 2 regarding propofol addiction. *p < 0.05, *p <* 0.01 and *p <* 0.001 and ***p < 0.0001.

In this study, 473 propofol action targets and 588 addiction targets were obtained by database screening, and Venn diagram analysis showed that 37 targets were shared between the two (Figure 4B). These targets were collated and visualized by a protein–protein interaction (PPI) network constructed from the STRING database and using Cytoscape 3.9.0 software (Figure 4C), with node color shades indicating the strength of the protein–protein interactions. The GO enrichment analysis identified 143 annotated pathways for biological processes, 51 cellular components and 56 molecular functions, and the analyses of each class The top 10 pathways were visualized (Figure 4D), involving multiple key biological processes such as signal transduction, chemical synaptic transmission, transmembrane ion transport, etc. KEGG pathway enrichment analysis screened out 23 signaling pathways, of which the top 10 were mainly involved in GABAergic synapses, morphine addiction, taste transmission, etc. (Figure 4E), and the neuroactive ligand-receptor interaction pathway was particularly prominent. Transcriptomics sequencing focused on pathways related to neurological ligand-active receptor interactions and cocaine addiction. Cross-analysis with transcriptomic data from cocaine addiction models, seen in dataset GSE108836 (29) identified 38 common targets of action (Figure 4F), and KEGG enrichment analysis further confirmed the importance of the neuroactive ligand-receptor pathway (Figure 5A). Combined with network pharmacological analysis, we screened the neuroactive receptor-ligand pathway and mapped the network of genes in the pathway, and found that the dopamine receptors DRD1 and DRD2 had a high degree of interactions (Figure 5B); therefore, molecular docking analyses of dopamine receptors DRD1 and DRD2 were performed, and the results showed that isoproterenol binds with binding energies of −6.9 and − 6.6 to DRD1 and DRD2, respectively (Figures 5C,D).

### 3.7 qPCR validation of differential genes

We screened eight differential genes from key pathways involved in neural ligand-receptor interactions for real-time fluorescence quantitative qPCR validation. These genes included DRD1, DRD2, TH, TRH, PPP1R1B, CHAT, RGS9, and GPR6. qPCR validation showed that the expression changes of these genes in the hippocampal region were consistent with the transcriptome sequencing results, thus validating the accuracy of the transcriptome data. This result is detailed in Figure 5E, which provides a solid experimental basis for further exploring the molecular mechanism of propofol addiction.

### 3.8 Western blot to verify the expression of dopamine receptor

In order to verify the expression of dopamine receptors in the hippocampus, protein immunoblotting was performed for verification, and the results showed that the expression of dopamine DRD1 and DRD2 in the hippocampus of mice in the propofol and Alcohol groups was decreased compared with that in the Saline group, which was consistent with the results of the sequencing analysis (Figures 5F,G).

## 4 Conclusion

Through this study, it was found that propofol had mental dependence, and the addictive effect was the highest at the dose of 75 mg/kg, and the addictive effect decreased after a certain dose, which provided a basis for the future use of propofol. Transcriptome and network pharmacology showed that propofol addiction caused significant expression of neuroactive ligand receptor pathway, and the tricarboxylic acid cycle and alanine, aspartate, and glutamate pathways in the hippocampus were significantly up-regulated. At the same time, *Campylobacter*, *Bacteroidetes*, *Actinobacteria*, and *Verrucomicrobia* in the intestinal flora were significantly increased. Therefore, it is inferred that the increase of SCFAS in the gut interacts with the activity of dopamine neurotransmitters in the hippocampal fatty acid metabolism, amino acid metabolism and neuroactive receptor ligand pathway.

## 5 Discussion

In this study, we combined transcriptomic, metabolomic, and metagenomic analyses of the hippocampus to provide new insights into the molecular mechanisms of propofol abuse addiction. These findings reveal the central role of the neural ligand-receptor interaction pathway in propofol addiction, especially the changes of dopamine neurotransmitters in the neuroreceptor pathway, and provide new insights into the detailed mechanism of propofol addiction.

In the analysis of transcriptomics results, significant changes in gene expression in the hippocampus following propofol addiction were observed, and these changes were mainly focused on the neuroactive ligand receptor pathway. Specifically, we found significant changes in the expression of the dopamine receptors DRD1 and DRD2, GPR6, and RGS9, which are closely related to neuroadaptive changes, synaptic plasticity, signaling, and other functions. These findings echo the neurobiological model proposed by Koob and Le Moal et al., which posits that addiction is a vicious cycle driven by a decline in the function of the brain reward system and activation of the anti-reward system, where chronic drug exposure leads to a decline in the function of the reward neurotransmitter system and concomitant activation of the anti-reward system, which induces down-regulation of dopamine receptor expression, thereby increasing the risk of drug craving and relapse (30, 31), causing downregulation of dopamine receptor expression and a high risk of drug craving and relapse. In addition, GPR6, a G protein-coupled receptor, has been identified as a novel therapeutic molecular target for cannabidiol, which provides new therapeutic perspectives (32), RGS9 and its specific splice variant RGS9-2 play roles in the regulation of morphine reward and dependence (33). Studies of the neuroactive ligand pathway have revealed that the pathway contains a variety of neurotransmitter systems including dopamine, endorphins, glutamate, norepinephrine, 5-hydroxytryptamine, and gamma-aminobutyric acid. Among these systems, the dopamine system plays a central role in the regulation of motor, emotional, and reward-related behaviors. Therefore, we believe that the reciprocal regulation of neural ligand-receptor interactions between genes such as DRD1, DRD2, GPR6, and RGS9 is closely linked to the reward effects and addictive behaviors of propofol.

Metabolomics further analyzes the effects of propofol addiction on the hippocampal region, which produces metabolites including citric acid, lysophosphatidylcholine, lysophosphatidylethanolamine, methylcoumarin, and docosahexaenoic acid, all of which are involved in cellular signaling, and energy metabolism related to the hippocampus. Especially dominated by citric acid, which breaks down into a variety of short-chain fatty acids, and the citric acid cycle also produces a variety of short-chain fatty acids. Current research suggests that short-chain fatty acids have anti-inflammatory effects, are involved in G protein-coupled receptors, neurotransmitter synthesis, neuroprotection, and the brain-gut axis (34), an increase in neuroprotection, signaling, and energy metabolism was found in the chemical classification of metabolites. In addition, significant cell membrane metabolic markers found in animal models of nicotine addiction and methamphetamine addiction were phosphatidylcholine (35, 36), and significant changes in energy metabolism-related metabolites such as citric acid cycle products and intermediates were similarly found in human serum as well as in the hippocampus of methamphetamine addicts (37), thus changes in metabolites in the hippocampal region of propofol addiction reflect the effects of propofol on energy metabolism pathways, neural signaling pathways involved in the production, activation, and functioning of neurotransmitters in the neural ligand-receptor interaction pathway to provide energetic substances, and reflect adaptive changes in neuronal cells in response to chronic exposure to propofol, which may further affect neurotransmitter function and neural network stability. This is consistent with existing findings.

In addition to transcriptomics and metabolomics, intestinal microbial macro-genomics sequencing was performed, and the results revealed that propofol addiction resulted in significant changes in the species abundance and composition of microorganisms in the intestine, such as a decrease in *Thick-walled phyla* and an increase in *Anopheles* and *Actinomycetes*, which were found to be the main phyla of the intestinal tract, and that the *Thick-walled phyla* and the *Anopheles* produce, by different means, SCFA, which can affect the brain by acting on G protein-coupled receptors expressed by cells in the intestine. For example, short-chain fatty acids act through G protein-coupled receptors such as FFAR2 and FFAR3, or by inhibiting histone deacetylase activity (38), and an increase in the *actinomycete phylum* improves host resistance to disease and maintains immune stability in the intestinal environment. Gut microbes can affect the immune system, including influencing the activation of immune cells and the production of cytokines. These cytokines can cross the blood–brain barrier and affect neuroinflammation and the activation state of microglia in the brain, which in turn affects neurotransmitter homeostasis. For example, anaerobic bacteria of the phylum *Actinobacteria* such as *Bifidobacteria*, *Propionibacteria*, *Corynebacteria*, and *Streptomyces* modulate the immune-inflammatory response by inducing regulatory T cells (39). In addition *Actinobacteria phylum* has the ability to produce antibiotics, which helps to inhibit the growth of pathogenic microorganisms, and is also involved in the synthesis of vitamins in the intestinal tract and maintenance of intestinal barrier function (40). Additionally the increase in *Lactobacillus* gates suggests that there may be vagal involvement in the brain-gut connection, and that certain specific gut microbes, such as *Lactobacillus rohita*, can transmit signals to the microbe-gut-brain axis via the vagus nerve, thereby affecting neuroendocrine metabolism and altering neurotrophic proteins, neurotransmitters in the hippocampus (41, 42). The gut microbiota communicates bi-directionally with the brain via the gut-brain axis. For example, changes in the metabolism of tryptophan, a precursor for the synthesis of the neurotransmitter 5-hydroxytryptophan, may affect mood and behavior (43). The mechanisms by which the gut microbiota influences mood and behavior through the gut-brain axis are multifaceted and involve complex interactions between the nervous, endocrine and immune systems.

These findings further underscore the pivotal role of the brain-gut axis in drug addiction and elucidate the intricate regulatory mechanisms between gut microbiota and hippocampal neural function. A key innovation of this study lies in the selection of propofol, a widely used clinical anesthetic, as the research subject, combined with neuroomics and gut microbiome analysis to explore the molecular mechanisms underlying drug addiction. However, we acknowledge several limitations, including the constrained scope of experimental data and the absence of large-scale dataset validation, which may restrict the generalizability and long-term applicability of our findings. Moreover, the precise mechanisms governing the interactions between gut microbiota and the host nervous system remain to be fully elucidated. Future studies should employ larger sample sizes and longitudinal designs to comprehensively unravel the molecular mechanisms of propofol addiction, thereby providing a more robust theoretical foundation and practical insights for clinical interventions.

## Data Availability

The original contributions presented in the study are publicly available. This data can be found here: Transcriptomics data can be found at the NCBI database under the accession number: BioProject: PRJNA1237536. Metagenomics data has also been uploaded to the NCBI database with the accession number: PRJNA1237534. Metabolomics data has been submitted to The MetaboLights Team, EMBL-EBI, with the project ID: MTBLS12337. Further inquiries can be directed to the corresponding author/s.

## Footnotes

^1^ https://www.bioinformatics.com.cn/

^2^ https://cadd.labshare.cn/cb-dock2/php/index.php
