# Supplementary figures and images for: Unraveling the mechanisms of propofol-induced psychological dependence: a multi-omics approach linked to gut microbiota in hippocampal function
Source: Front Med (Lausanne). 2025 Apr 3;12:1539467. doi: 10.3389/fmed.2025.1539467 (PMC12005058; doi:10.3389/fmed.2025.1539467)

DRD1

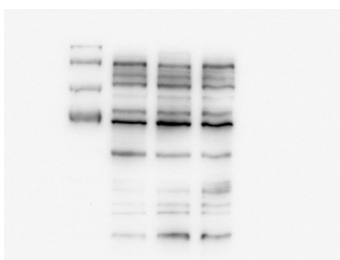

-actin

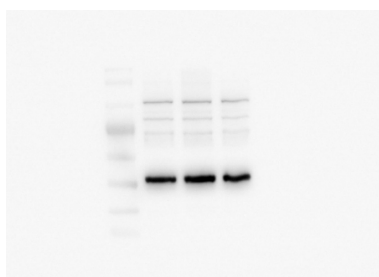

DRD2

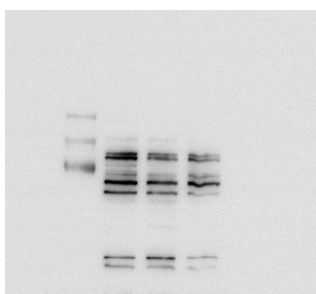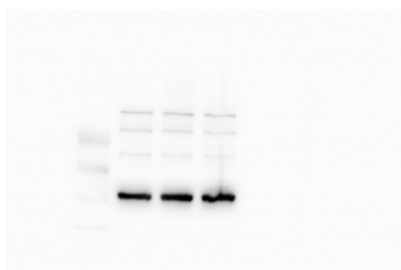

Supplement: Supplementary file 1 [file Data_Sheet_1.zip › raw data/WB.pdf]
