# Supplementary material for: Unraveling the mechanisms of propofol-induced psychological dependence: a multi-omics approach linked to gut microbiota in hippocampal function
Source: Front Med (Lausanne). 2025 Apr 3;12:1539467. doi: 10.3389/fmed.2025.1539467 (PMC12005058; doi:10.3389/fmed.2025.1539467)
Supplement: Supplementary file 1 [file Data_Sheet_1.zip › raw data/R代码.doc]

#Mapping gene-metabolite heat maps#

dx <- read.csv("dx.csv",header = T,row.names = 1)

gene <- read.csv("gene.csv",header = T,row.names = 1)

gene1 <- as.matrix(gene)

dx1 <- as.matrix(dx)

cor(dx1[(row.names='Docosahexanoic acid'),],gene1[(row.names='Penk'),])

gene2 <- t(gene1)

dx2 <- t(dx1)

library(WGCNA)

metaGeneCor.r <- cor(gene2, dx2, method="pearson")

write.csv(metaGeneCor.r,file="GeneCorMeta.csv")

nmeta=ncol(dx)

metaGeneCor.p <- corPvalueStudent(metaGeneCor.r, nmeta)

write.csv(metaGeneCor.p,file = "MetaCorGene.p.csv")

transposedMetaGeneCor.r <- t(metaGeneCor.r)

transposedSignificantMarkers <- t(significant_markers)

pheatmap(

transposedMetaGeneCor.r,

display_numbers = transposedSignificantMarkers,

show_rownames = TRUE, show_colnames = TRUE,

number_color = "black",

cellwidth = 10,

cellheight = 20,

fontsize = 8,

fontsize_row = 9,

fontsize_col = 9,

angle_col = 90,

border_color = "white",

main = "Gene-metabolite Heatmap" )

library(pheatmap)

pheatmap(metaGeneCor.r,show_rownames = F,show_colnames = F)

library(pheatmap)

pheatmap(metaGeneCor.r,

show_rownames = TRUE,

show_colnames = TRUE,

cellwidth = 20,

cellheight = 10,

fontsize = 10,

fontsize_row = 8,

fontsize_col = 8,

clustering_distance_rows = "euclidean",

clustering_method = "complete",

color = colorRampPalette(c("blue", "white", "red"))(100))

significant_markers <- ifelse(metaGeneCor.p < 0.05, "*", "")

library(pheatmap)

pheatmap(metaGeneCor.r,

display_numbers = significant_markers,

show_rownames = FALSE,

show_colnames = FALSE,

number_color = "black"

)

library(pheatmap)

pheatmap(

metaGeneCor.r,

display_numbers = significant_markers,

show_rownames = TRUE,

show_colnames = TRUE,

number_color = "black",

cellwidth = 25,

cellheight = 10,

fontsize = 7,

fontsize_row = 8,

fontsize_col = 8,

angle_col = 45,

border_color = "white",

main = "Correlation Heatmap" )

dev.off()
